# Supplementary material for: Functional cardiac fibroblasts derived from human pluripotent stem cells via second heart field progenitors
Source: Nat Commun. 2019 May 20;10:2238. doi: 10.1038/s41467-019-09831-5 (PMC6527555; doi:10.1038/s41467-019-09831-5)
Supplement: Supplementary file 1 — Supplementary Information [file 41467_2019_9831_MOESM1_ESM.pdf]

Functional Cardiac Fibroblasts Derived from Human Pluripotent Stem Cells via Second Heart Field Progenitors

Zhang et al.

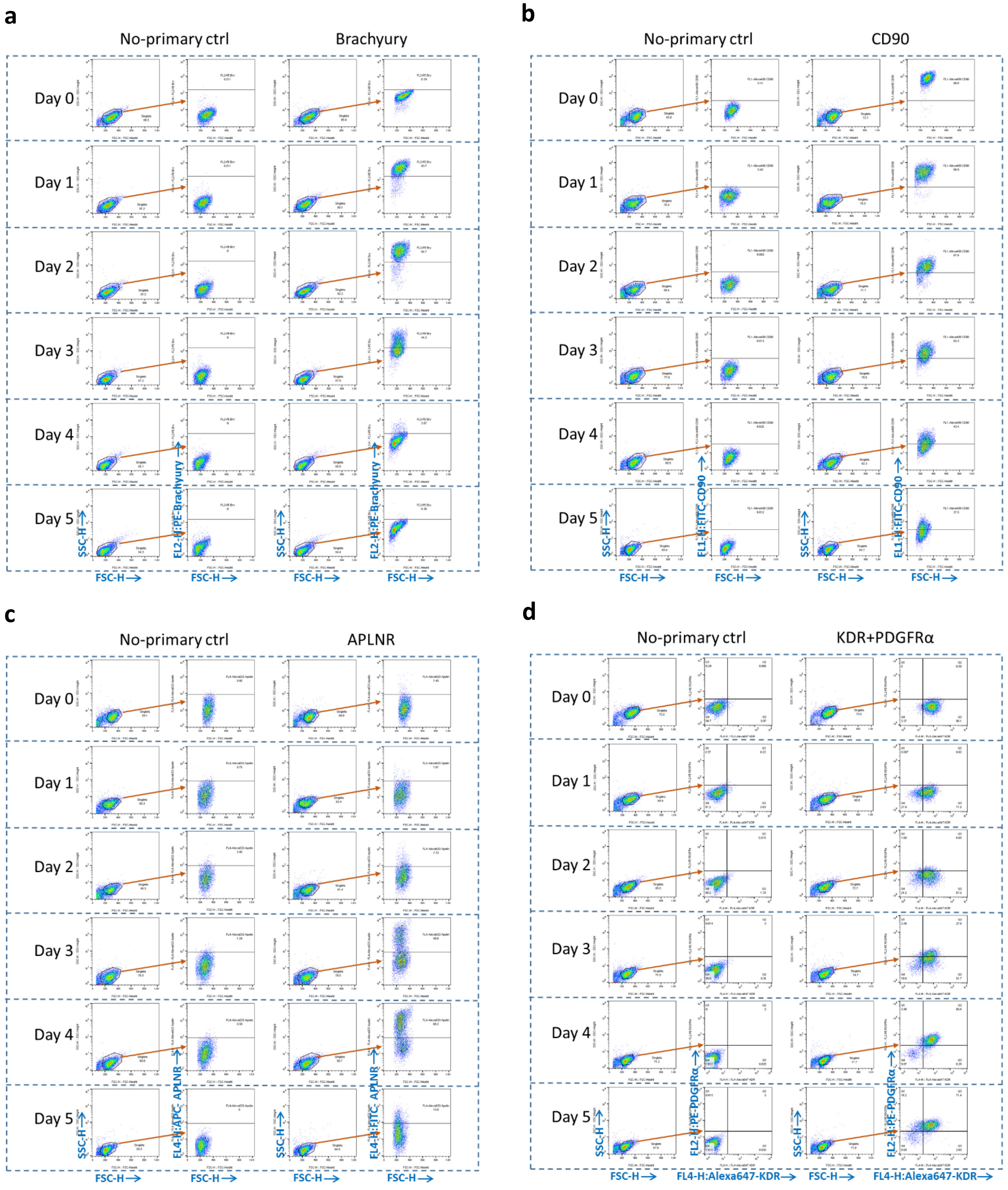

Supplementary Figure 1. Gating strategies used for flow cytometry presented in Fig. 1b to analyze cells expressing Brachyury **(a)**, CD90 **(b)**, APLNR **(c)** and KDR/PDGFRα **(d)**. No-primary ctrl and the corresponding antibody-labeled sample are gated the same. Data were collected on BD FACSCalibur.

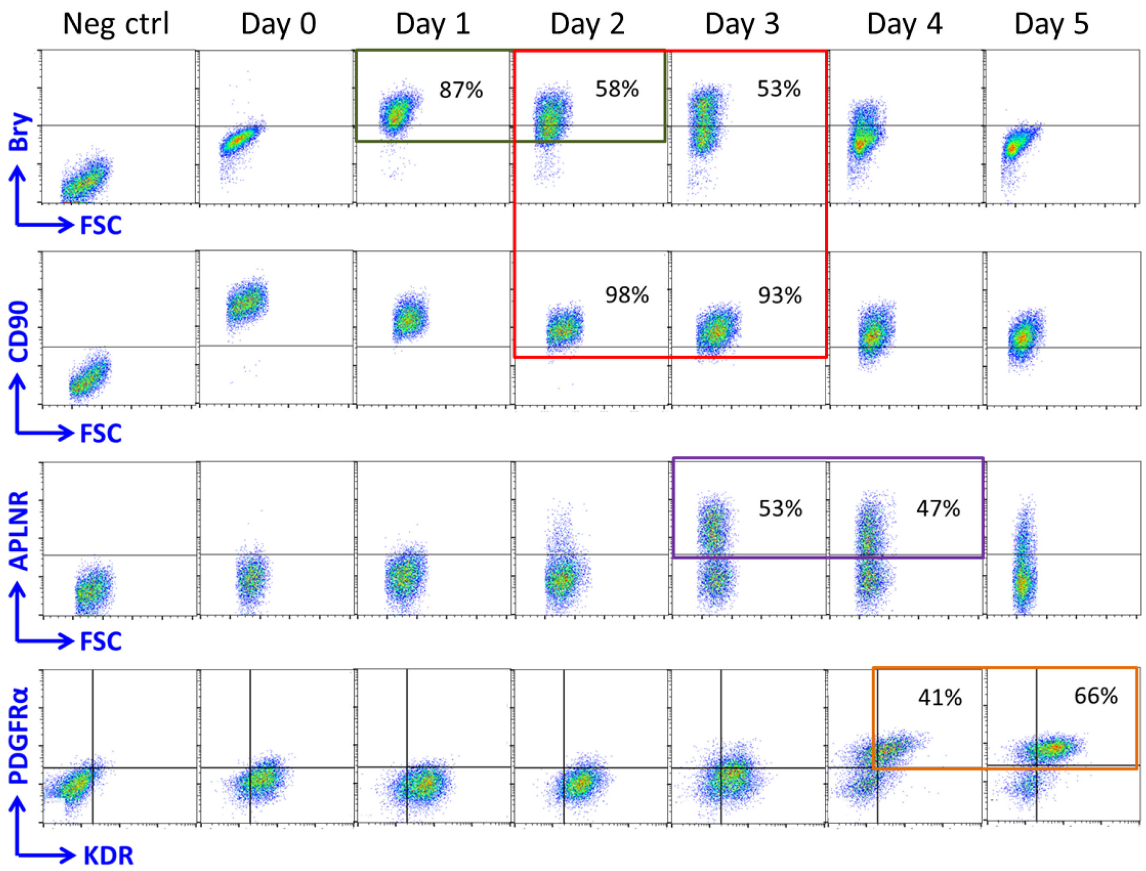

Supplementary Figure 2. Identification of progenitors in cardiac differentiation of the hESC line H1. Flow cytometry of stage-specific progenitors labeled by Brachyury (Bry), CD90, Apelin receptor (APLNR), PDGFRα and KDR in early differentiation (day 0-5) of the GiWi protocol. No-primary antibody controls and isotype controls were performed for each time point, and the day 0, no-primary antibody control (Neg ctrl) are shown as an example.

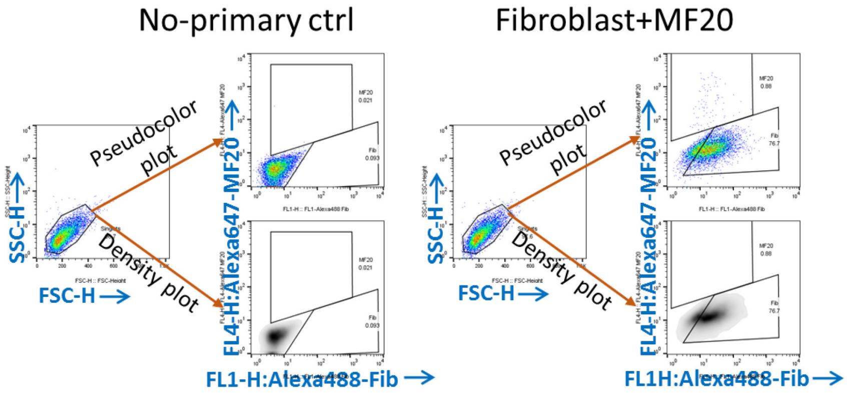

Supplementary Figure 3. Gating strategy used for flow cytometry presented in Fig. 2b to analyze cells expressing Fibroblast (clone TE-7) and MF20. No-primary ctrl and the corresponding antibody-labeled sample are gated the same. Data were collected on BD FACSCalibur.

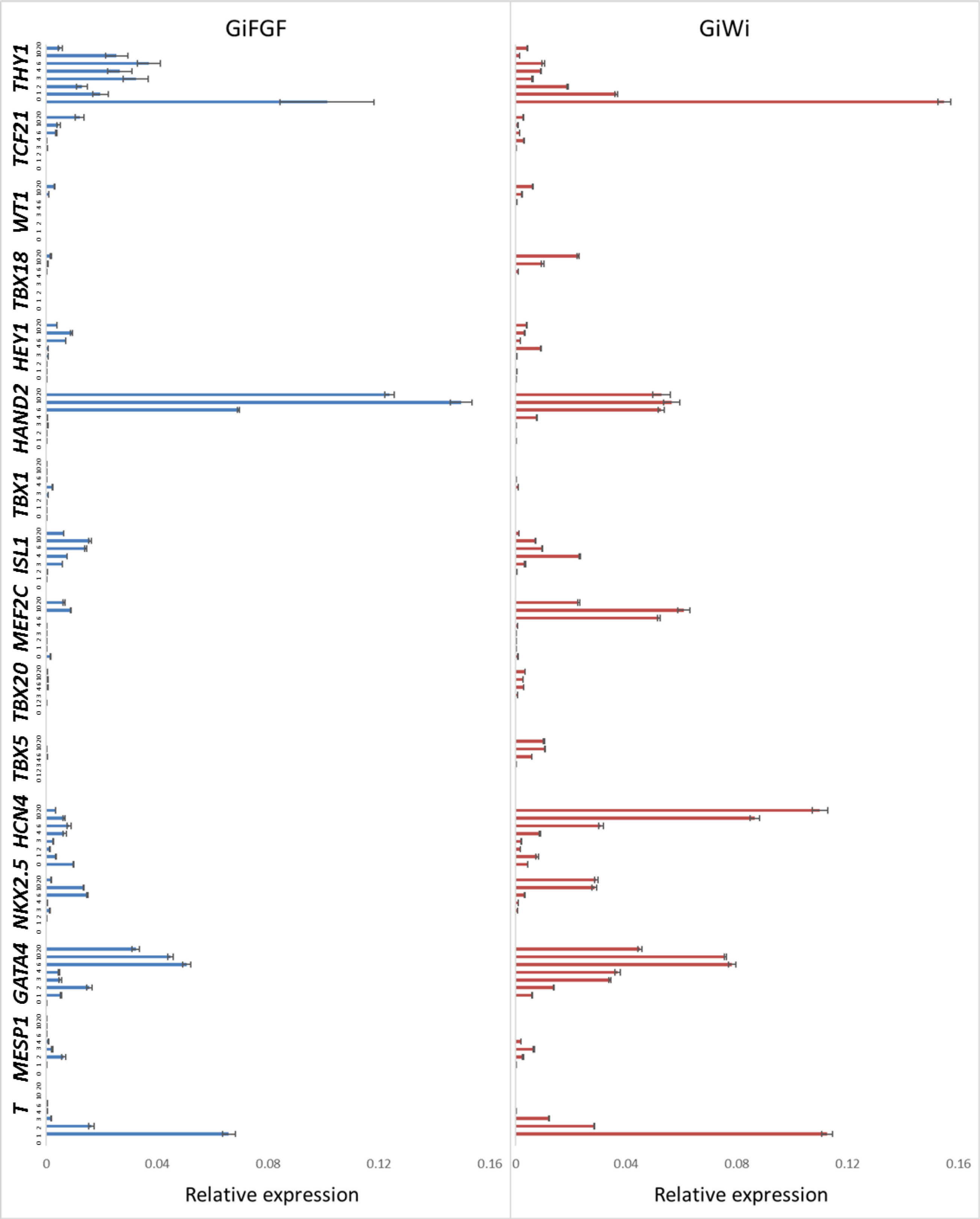
GiFGF
GiWi
THY1
TCF21
WT1
TBX18
HEY1
HAND2
TBX1
ISL1
MEF2C
TBX20
TBX5
HCN4
NKX2.5
GATA4
MESP1
T
0
0.04
0.08
0.12
0.16
Relative expression
Relative expression

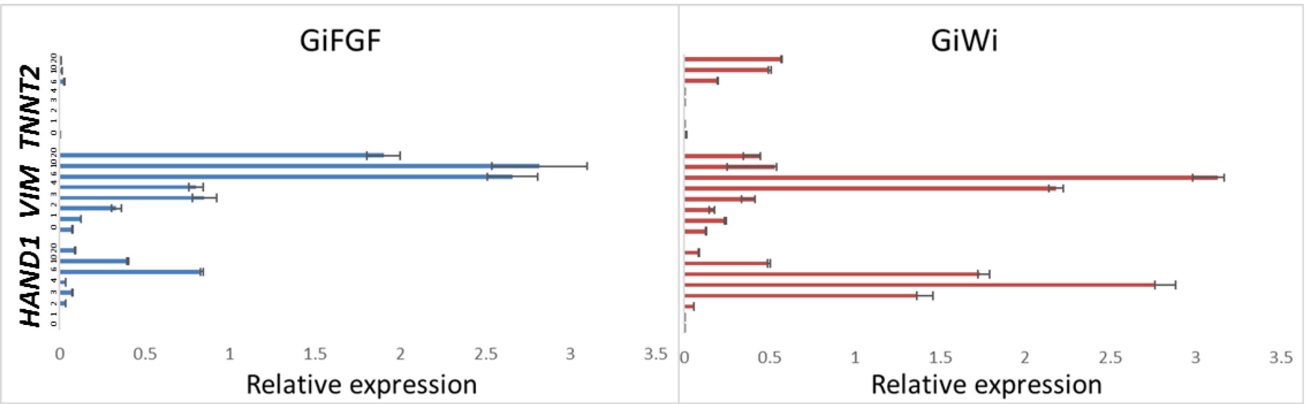

Supplementary Figure 4. Gene expression by qRT-PCR in the CF differentiation protocol – GiFGF, compared with the CM differentiation protocol – GiWi for relevant cardaic genes. qRT-PCR was performed on samples at the time indicated in both GiFGF and GiWi protocols with results plotted as relative expression normalized to *GAPDH*. N=3 technical replicates. Data are mean ± SEM. The data are from hiPSC line DF19-9-11T.

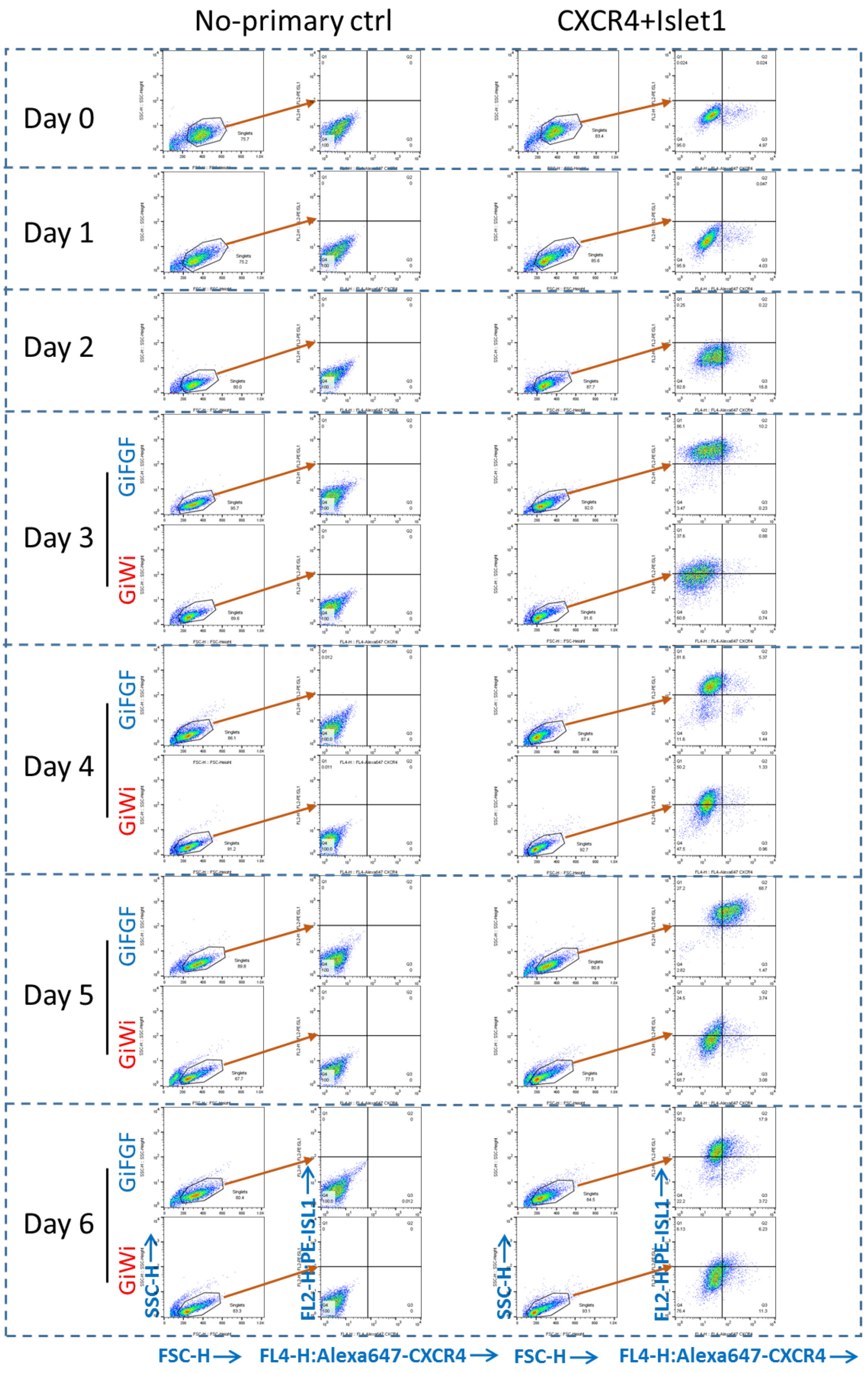

Supplementary Figure 5. Gating strategy used for flow cytometry presented in Fig. 4a to analyze cells expressing CXCR4 and Islet1. No-primary ctrl and the corresponding antibody-labeled sample are gated the same. Data were collected on BD FACSCalibur.

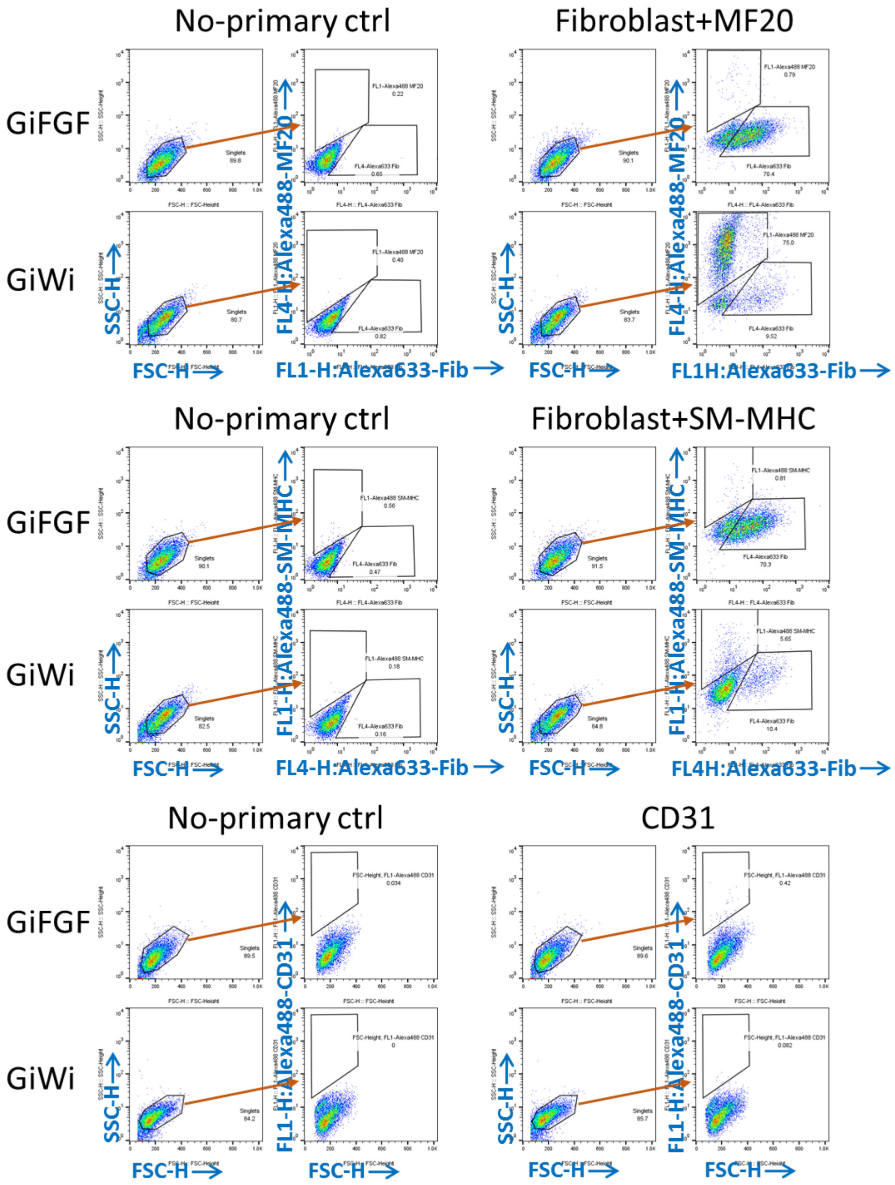

Supplementary Figure 6. Gating strategies used for flow cytometry presented in Fig. 4b to analyze cells expressing Fibroblast (clone TE-7), MF20, smooth muscle myosin heavy chain (SM-MHC) and CD31. No-primary ctrl and the corresponding antibody-labeled sample are gated the same. Data were collected on BD FACSCalibur.

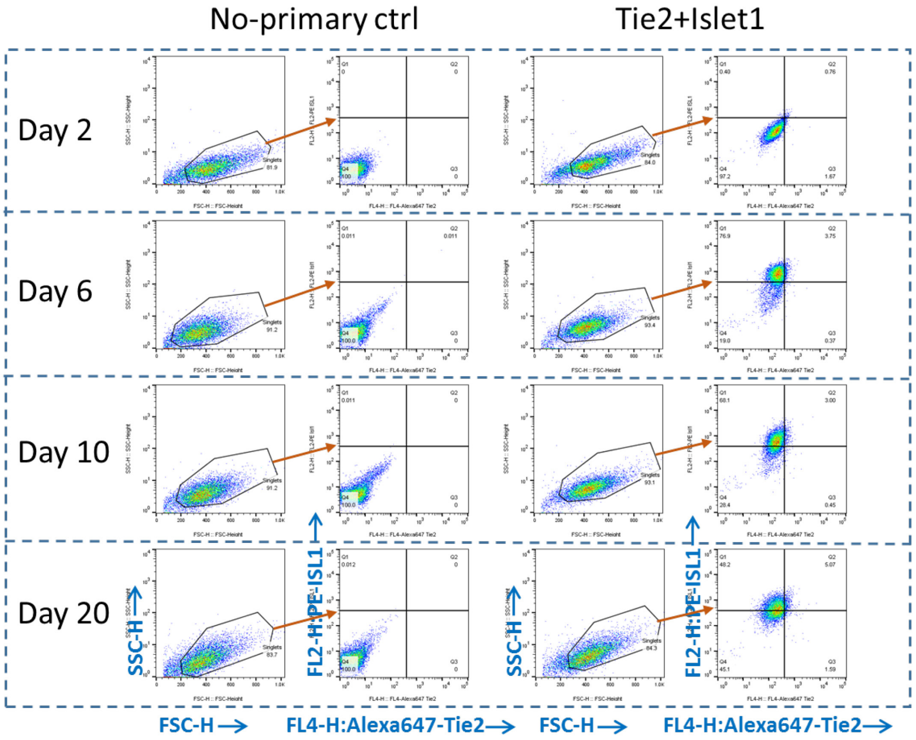

Supplementary Figure 7. Gating strategy used for flow cytometry presented in Fig. 4c to analyze cells expressing Tie2 and Islet1. No-primary ctrl and the corresponding antibody-labeled sample are gated the same. Samples from day 2, 6, 10 and 20 are shown as an example. Data were collected on BD FACSCalibur.

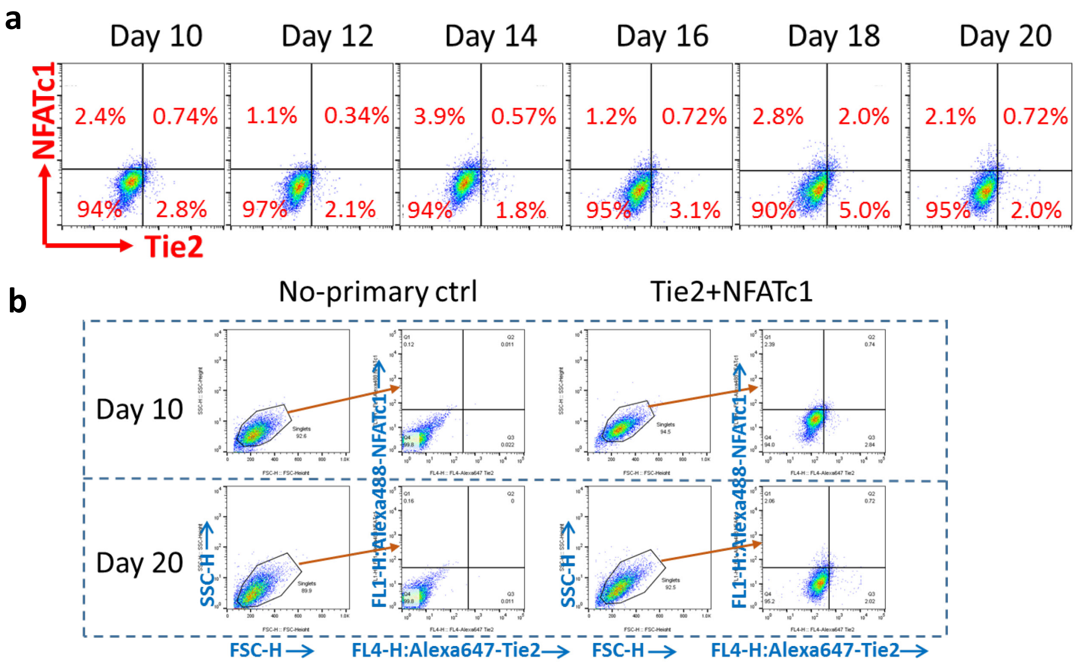

Supplementary Figure 8. Flow cytometry analysis for NFATc1 and Tie2 in the GiFGF protocol. **(a)** Cells differentiated from DF19-9-11T hiPSCs from day 10 to 20 of the protocol were co-labeled for the endocardial transcription factor, NFATc1, and the endothelial progenitor marker, Tie2 and were measured by flow cytometry. **(b)** Gating strategy used for the flow cytometry in (a). No-primary ctrl and the corresponding antibody-labeled sample are gated the same. Samples from day 10 and 20 are shown as an example. Data were collected on BD FACSCalibur.

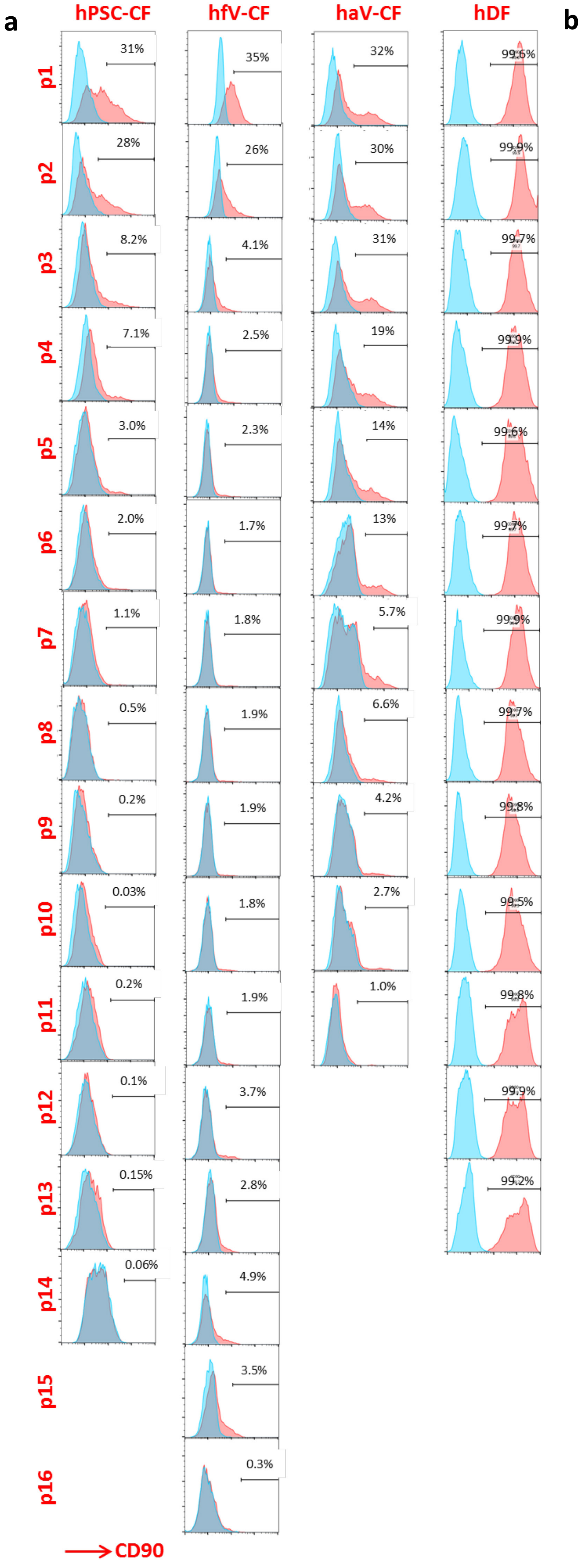

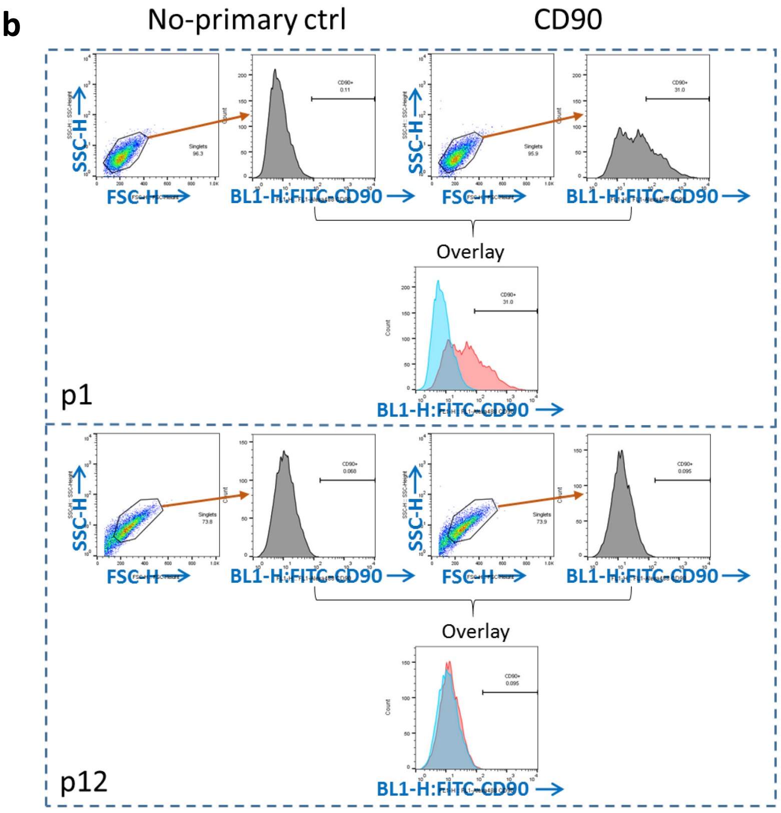

Supplementary Figure 9. Flow cytometry analysis of CD90 expression in hPSC-CFs, hfV-CFs, haV-CFs and hDFs during passaging. **(a)** Histogram plot for CD90 expression (pink) over isotype control (blue) of hPSC-CFs, hfV-CFs, haV-CFs and hDFs. The hPSC-CFs are derived from hiPSC line DF9-9-11T. The hfV-CFs are from Cell Applications, Inc. The haV-CFs are from Lonza, NHCF-V. The hDFs are from 020a line. **(b)** Gating strategy used for the flow cytometry in (a). No-primary ctrl and the corresponding antibody-labeled sample are gated the same. Passage 1 and 12 of the hPSC-CFs are shown as an example. Data were collected on BD FACSCalibur.

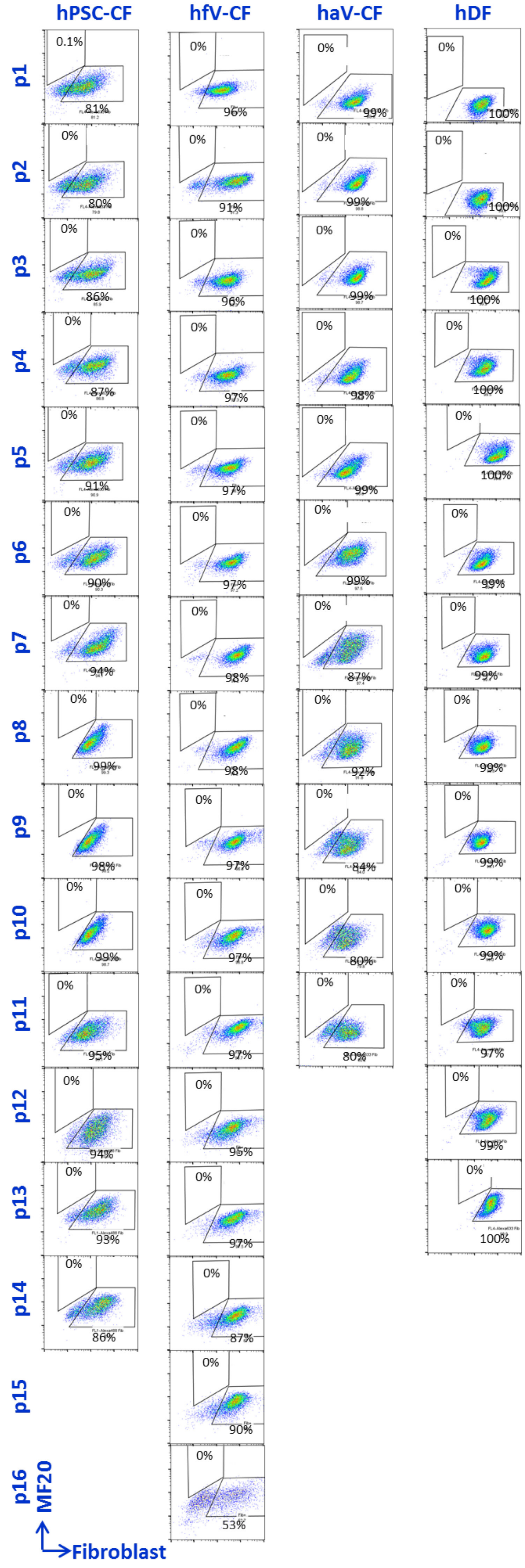

Supplementary Figure 10. Flow cytometry analysis of makers expression for fibroblasts (anti-human fibroblast, clone TE-7) and cardiomyocytes (MF20) in hPSC-CFs, hfV-CFs, haV-CFs and hDFs during passaging. Pseudocolor plot for expression of Fibroblast and MF20. The hPSC-CFs are derived from hiPSC line DF9-9-11T. The hfV-CFs are from Cell Applications, Inc. The haV-CFs are from Lonza, NHCF-V. The hDFs are from 020a line. Gating strategy used for the flow cytometry are presented in Supplementary Figure 3.

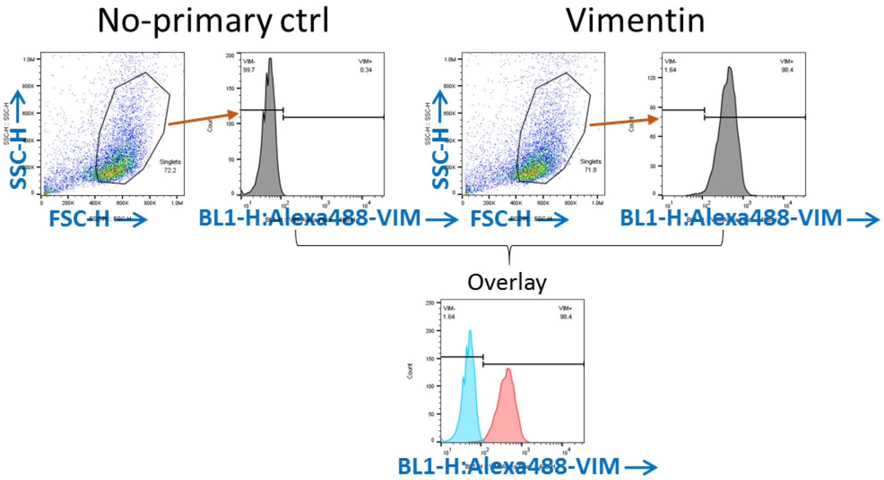

Supplementary Figure 11. Gating strategy used for flow cytometry presented in Fig. 6a. No-primary ctrl and the corresponding antibody-labeled sample are gated the same. The analysis for the hPSC-CF sample are shown as an example. Data were collected on BD FACSCalibur.

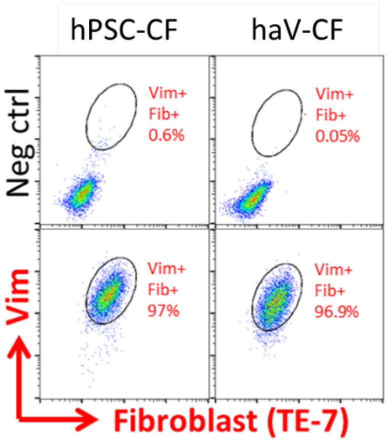

Supplementary Figure 12. Flow cytometry analysis of cells co-labeled by the antibodies of fibroblast (clone TE-7) and vimentin in the hPSC-CFs and haV-CFs. Neg ctrl was no-primary antibody control. The hPSC-CFs are derived from hiPSCs DF19-9-11T and haV-CF are from Lonza, NHCF-V.

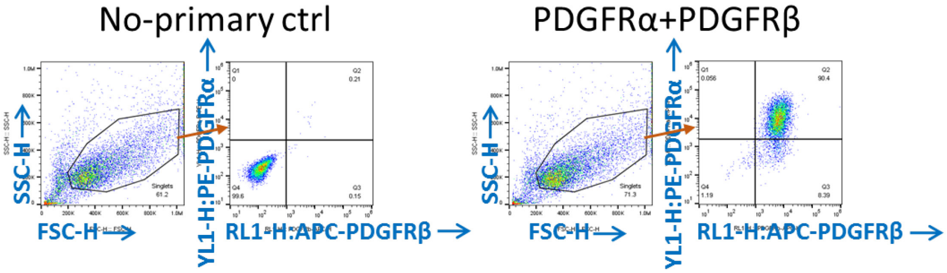

Supplementary Figure 13. Gating strategy used for flow cytometry presented in Fig. 6b. No-primary ctrl and the corresponding antibody-labeled sample are gated the same. The analysis for the hPSC-CF sample are shown as an example. Data were collected on ThermoFisher Attune Nxt.

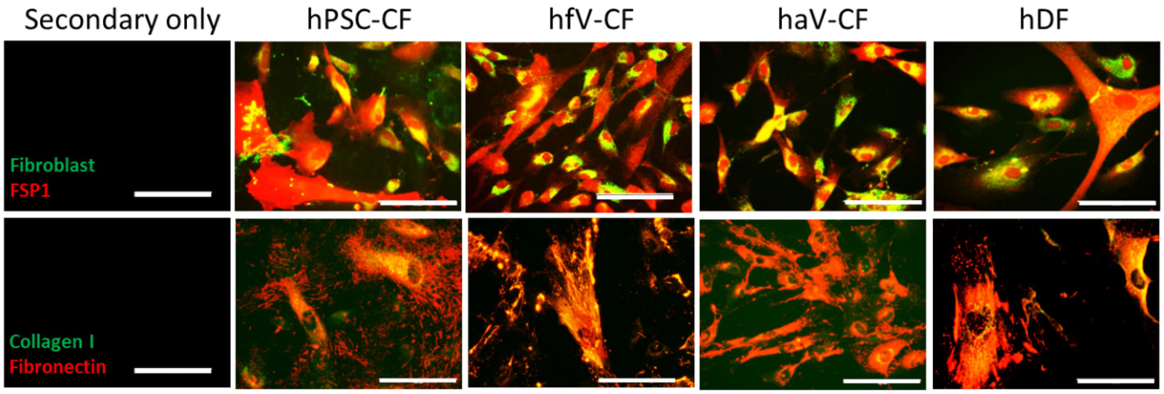

Supplementary Figure 14. Immunolabeling by antibodies for fibroblast (clone TE-7), fsp1, collagen I and fibronectin in hPSC-CFs, hfV-CFs, haV-CFs and hDFs. Scale bars are 100 μm. All fibroblasts samples used in the measurement are from passages 3-7. The hPSC-CFs are derived from hiPSC line DF-19-9-11T. The primary hfV-CFs are from Cell Applications, Inc. The haV-CFs are from Lonza, NHCF-V. The hDFs are from 023a line.

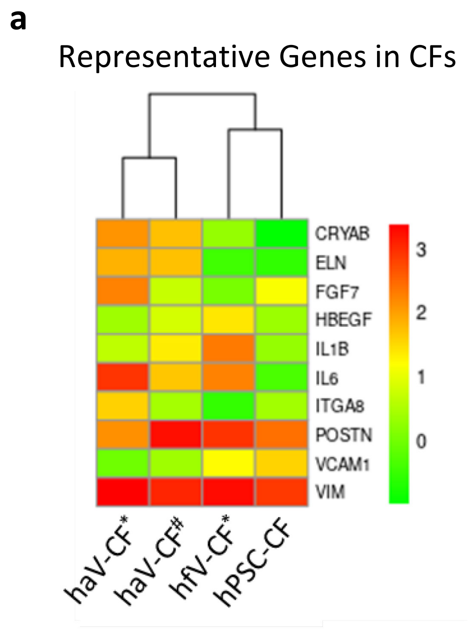

Supplementary Figure 15. Comparison of gene expression in hPSC-CFs with human fetal and adult cardiac fibroblasts including the data of Jonnson et al.[1] **(a)** The 10 representative, differentially expressed genes[1] in human fetal and adult cardiac fibroblasts. **(b)** The 58 cardiac factors expressed in CFs. **(c)** ECM gene expression in CFs. RNA-seq data of hPSC-CF, hfV-CF and haV-CF are compared. The heatmap of gene expression is organized by unsupervised cluster analysis. The hPSC-CFs are derived from hiPSCs DF19-9-11T. The primary hfV-CFs* and haV-CFs* are from Cell Applications, Inc. as published in Jonsson et al.[1]. The primary haV-CFs# are from Lonza, NHCF-V. The fpkm (fragments per kilobase of exon per million fragments mapped) values from the RNA-seq across samples were used for the analysis.

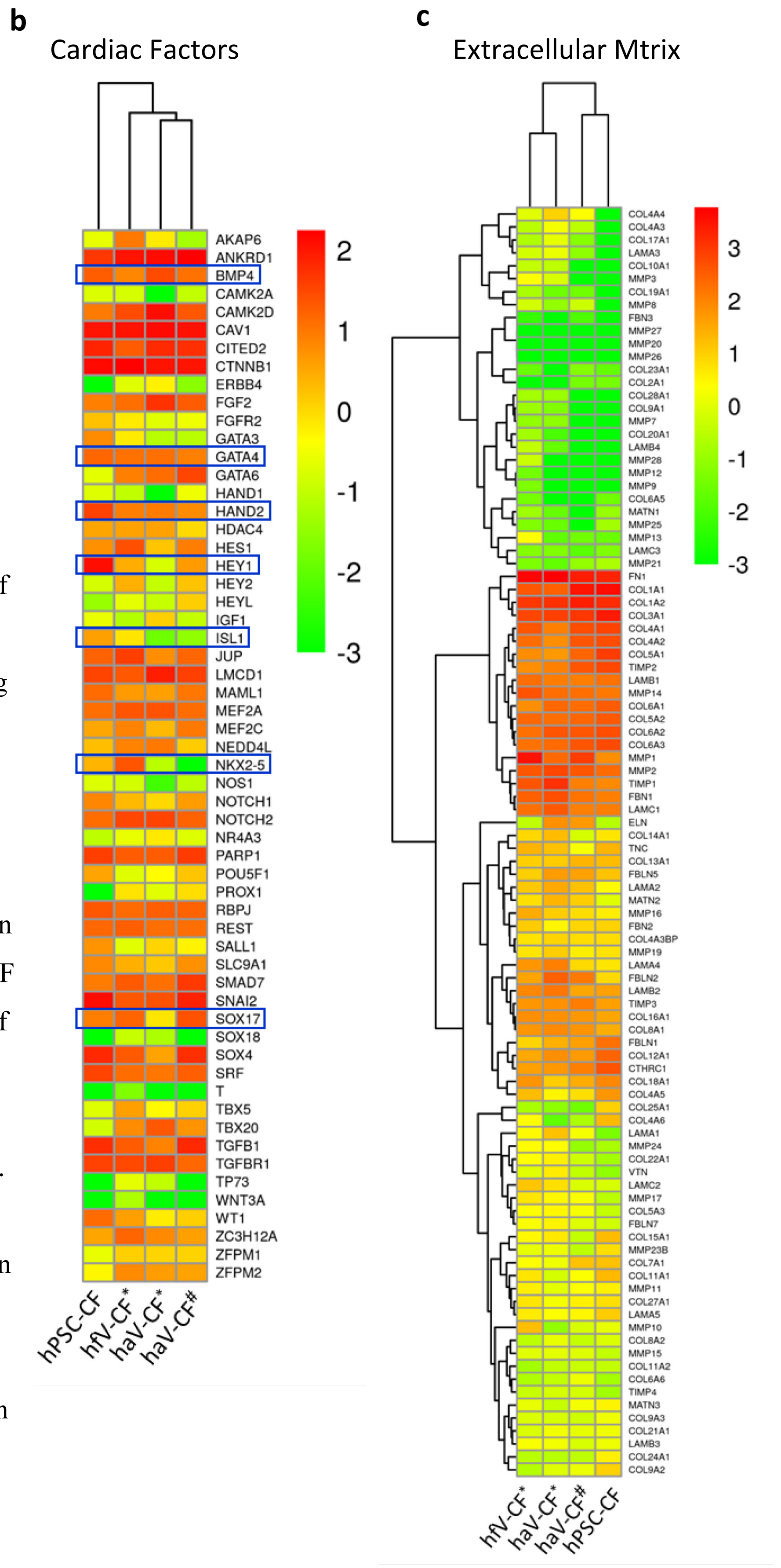

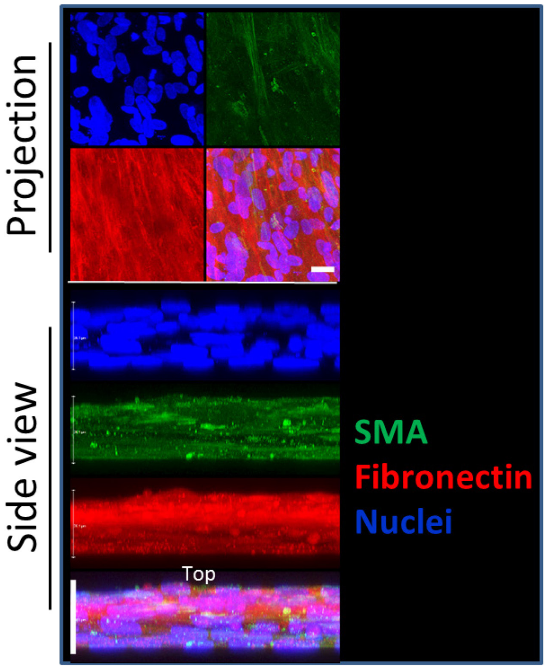

Supplementary Figure 16. Immunolabeling for fibronectin and α-smooth muscle actin in high density, multilayer hPSC-CF culture. Confocal imaging of permeabilized, high density 3D culture of hPSC-CFs immunolabeled by fibronectin and α-smooth muscle actin (SMA) antibodies. Z-scans are presented as projection images in the top panel, scale bar is 25 μm, and 3D reconstructions showing side views in lower panel, scale bar is 40 μm. Please see Supplementary Movie 5 for complete series of Z-scan images.

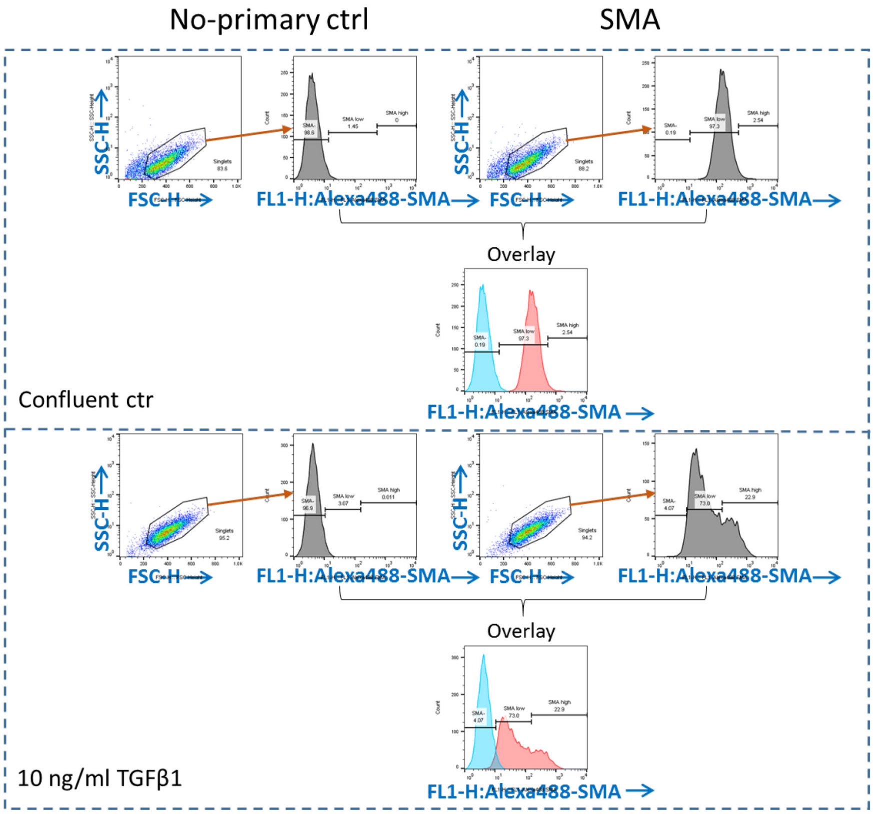

Supplementary Figure 17. Gating strategy used for flow cytometry presented in Fig. 9. No-primary ctrl and the corresponding antibody-labeled sample are gated the same. The analysis for the hPSC-CF sample are shown as an example. Data were collected on BD FACSCalibur.

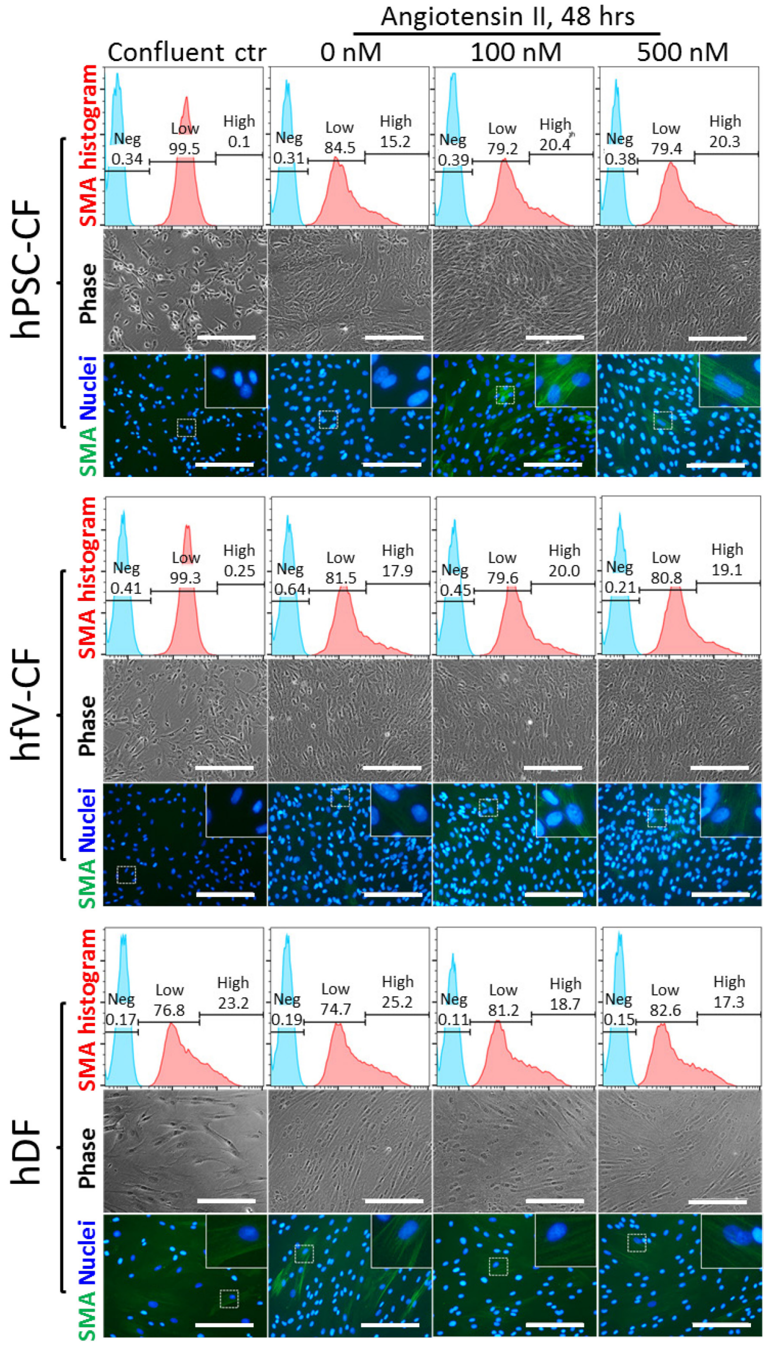

Supplementary Figure 18.

Angiotensin II induced myofibroblast transformation. Analysis of α-smooth muscle actin (SMA) expressing cells to assay for myofibroblast transformation from hPSC-CF, hfV-CF and hDF cultures. All fibroblast cultures (passages 4-6) were treated with Ang II (100 nM and 500 nM) for 48 hrs in DMEM+10%FBS medium. Expression of SMA was measured by both flow cytometry (upper panel) and immunolabeling (lower panel). Phase contrast images of representative cultures are shown in the middle panel. Scale bars are 200 µm. The hPSC-CFs are derived from DF19-9-11T hiPSCs. The hfV-CFs are from Cell Applications, Inc. and the hDFs are from 023a line. Gating strategy used for the flow cytometry are presented in Supplementary Figure 17.

Supplementary Table 1. Formulation of cardiac fibroblast differentiation basal medium (CFBM)

Components	Final concentration
DMEM, high glucose (4.5g/L)	basal medium
HLL Supplement: HSA (human serum albumin), linoleic acid and lecithin	HSA: 500 µg/mL<br>Linoleic Acid: 0.6 µM<br>Lecithin: 0.6 µg/mL
Ascorbic Acid	50 µg/mL
GlutaMAX	7.5 mM
Hydrocortisone Hemisuccinate	1.0 µg/mL
rh Insulin	5 µg/mL

Supplementary Table 2. Primary antibodies used in flow cytometry (FC) and immunocytochemistry (ICC)

Human/Mouse Brachyury PE-conjugated	R&D (IC2085P)	Goat	IgG	10ul/1x10^6 cells (FC)
FITC anti-human CD90	Biolegend (328108)	Mouse	IgG_1	5ul/1x10^6 cells (FC)
Human APLNR APC-conjugated	R&D (FAB856A)	Mouse	IgG_3	3ul/1x10^6 cells (live labeling) (FC)
Alexa Fluor 647 Mouse Anti-Human CD309 (KDR)	BD (560495)	Mouse	IgG_1	20ul/1x10^6 cells (FC)
PE Mouse Anti-Human CD140a (PDGFRα)	BD (556002)	Mouse	IgG_{2a}	20ul/1x10^6 cells (FC)
APC-anti-human CD140b (PDGFRβ)	Biolegend (323608)	Mouse	IgG_1	5ul/1x10^6 cells (FC)
FITC Mouse Anti-Human CD31	BD (560984)	Mouse	IgG_1	20ul/1x10^6 cells (FC)
PE Mouse anti-Islet-1	BD (562547)	Mouse	IgG_1	20ul/1x10^6 cells (FC)
Human CXCR4 APC-conjugated Antibody	R&D (FAT173a)	Mouse	IgG_{2b}	10ul/1x10^6 cells (FC)
Alexa Fluor 647 anti-human CD202b (Tie2/Tek) Antibody	Biolegend (334210)	Mouse	IgG_1	5ul/1x10^6 cells (FC)
Alexa Fluor 488 anti-NFATc1 Antibody	Biolegend (649604)	Mouse	IgG_1	0.5ul/1x10^6 cells (FC)
Mouse Anti-Human Fibroblasts (Clone TE-7)	CBL271	Mouse	IgG_1	1:100 dilution (FC, ICC)
MF20	DSHB	Mouse	IgG_{2b}	1:20 dilution (FC, ICC)
Troponin T, Cardiac Isoform Ab-1 (Clone 13-11)	ThermoFisher Scientific (#MS-295-P)	Mouse	IgG_1	1:200 dilution (FC, ICC)
Anti-Smooth Muscle Myosin	Biomedical Technologies Inc. (BT-562)	Rabbit	IgG	1:500 dilution (FC, ICC)
GATA4 (H-112)	Santa Cruz (sc-9053)	Rabbit	IgG	1:200 dilution (ICC)
Anti-Fibroblast-specific Protein 1 (S100A4)	EMD Millipore (#ABF32)	Rabbit	IgG	1:500 dilution (ICC)
Fibronectin (A-11)	Santa Cruz (sc-271098)	Mouse	IgG_{2b}	1:100 dilution (ICC)
COL1A (COL-1)	Santa Cruz (sc-59772)	Mouse	IgG_1	1:100 dilution (ICC)
Anti-Periostin	Abcam (ab14041)	Rabbit	IgG	1:500 dilution (FC, ICC)
Human/Mouse/Rat Vimentin Alexa Fluor® 488-conjugated, Clone # 280618	R&D (IC2105G)	Rat	IgG_{2a}	5 μL/1x10^6 cells (FC)
Actin, Smooth Muscle Ab-1 (Clone 1A4; same as asm-1)	ThermoFisher Scientific (MS-113-P)	Mouse	IgG_{2a}	1:100 dilution (FC, ICC)
Mouse IgG2a kappa Isotype Control, PE	eBioscience (12-4724-42	Mouse	IgG_{2a}	5ul/1x10^6 cells (FC)
Alexa Fluor 647 Mouse IgG1, k Isotype Ctrl (FC) Antibody	Biolegend (400130)	Mouse	IgG_1	5ul/1x10^6 cells (FC)
Alexa Fluor 488 Mouse IgG1 k Isotype Control	BD (557782)	Mouse	IgG_1	20ul/1x10^6 cells (FC)
FITC Mouse IgG2a, k Isotype Control	BD (556652)	Mouse	IgG_1	20ul/1x10^6 cells (FC)
Alexa Fluor 647 Mouse IgG2b k Isotype Control	BD (557903)	Mouse	IgG_1	5ul/1x10^6 cells (FC)
Mouse IgG3 Isotype Control-APC	R&D (IC007A)	Mouse	IgG_3	5ul/1x10^6 cells (FC)

Supplementary Table 3. Primers for quantitative RT-PCR

Genes	TaqMan® Gene Expression Assay ID
GAPDH	Hs99999905_ml
T	Hs00610080_ml
MESP1	Hs00251489_ml
GATA4	Hs00171403_ml
NKX2-5	Hs00231763_ml
HCN4	Hs00975492_ml
TBX5	Hs01052563_ml
TBX20	Hs00396596_ml
MEF2C	Hs00231149_ml
ISL1	Hs00158126_ml
TBX1	Hs00271949_ml
HAND1	Hs02330376_s1
HAND2	Hs00232769_ml
HEY1	Hs01114113_ml
TBX18	Hs01385457_ml
WT1	Hs01103751_ml
TCF21	Hs00162646_ml
SNAIL1	Hs00195591_ml
SNAIL2	Hs00950344_ml
THY1	Hs00264235_s1
OCT4	Hs01895061_u1
VIM	Hs00185584_ml
TNNT2	Hs00165960_ml
POSTN	Hs01566750_ml
DDR2	Hs01025953_ml
PECAM1	Hs01065279_ml
MYH11	Hs00224610_ml

Supplementary References

1. Jonsson, M.K.B. *et al.* A Transcriptomic and Epigenomic Comparison of Fetal and Adult Human Cardiac Fibroblasts Reveals Novel Key Transcription Factors in Adult Cardiac Fibroblasts. *JACC Basic Transl Sci* **1**, 590-602 (2016).
