# Supplementary material for: Functional cardiac fibroblasts derived from human pluripotent stem cells via second heart field progenitors
Source: Nat Commun. 2019 May 20;10:2238. doi: 10.1038/s41467-019-09831-5 (PMC6527555; doi:10.1038/s41467-019-09831-5)
Supplement: Supplementary file 3 — Reporting Summary [file 41467_2019_9831_MOESM3_ESM.pdf]

nature research

Corresponding author(s): Timothy J. Kamp

Last updated by author(s): Mar 19, 2019

Reporting Summary

Nature Research wishes to improve the reproducibility of the work that we publish. This form provides structure for consistency and transparency in reporting. For further information on Nature Research policies, see Authors & Referees and the Editorial Policy Checklist.

Statistics

For all statistical analyses, confirm that the following items are present in the figure legend, table legend, main text, or Methods section.

n/a	Confirmed	
☐	☒	The exact sample size (n) for each experimental group/condition, given as a discrete number and unit of measurement
☐	☒	A statement on whether measurements were taken from distinct samples or whether the same sample was measured repeatedly
☐	☒	The statistical test(s) used AND whether they are one- or two-sided<br>*Only common tests should be described solely by name; describe more complex techniques in the Methods section.*
☐	☒	A description of all covariates tested
☐	☒	A description of any assumptions or corrections, such as tests of normality and adjustment for multiple comparisons
☐	☒	A full description of the statistical parameters including central tendency (e.g. means) or other basic estimates (e.g. regression coefficient) AND variation (e.g. standard deviation) or associated estimates of uncertainty (e.g. confidence intervals)
☐	☒	For null hypothesis testing, the test statistic (e.g. F, t, r) with confidence intervals, effect sizes, degrees of freedom and P value noted<br>*Give P values as exact values whenever suitable.*
☒	☐	For Bayesian analysis, information on the choice of priors and Markov chain Monte Carlo settings
☐	☒	For hierarchical and complex designs, identification of the appropriate level for tests and full reporting of outcomes
☒	☐	Estimates of effect sizes (e.g. Cohen's d, Pearson's r), indicating how they were calculated

Our web collection on statistics for biologists contains articles on many of the points above.

Software and code

Policy information about availability of computer code

Data collection	No customized computer programs were used for data collection.
Data analysis	Genes for specific functional groups were selected by searching online tool AmiGO2 (http://amigo.geneontology.org) using key words and manually confirmed. Heatmaps were generated using pheatmap_1.0.8 in R version 3.2.3. The similarity heatmap showed the Euclidean distances between samples (Fig. 7a) which were calculated using R dist function on normalized read counts. Gene ontology (GO) analysis using the online Functional Annotation Tool (DAVID Bioinformatics Resources 6.7, NIAID/NIH) for the differentially expressed genes (Fig. 7d, Supplementary Data 1, 2,).

For manuscripts utilizing custom algorithms or software that are central to the research but not yet described in published literature, software must be made available to editors/reviewers. We strongly encourage code deposition in a community repository (e.g. GitHub). See the Nature Research guidelines for submitting code & software for further information.

Data

Policy information about availability of data

All manuscripts must include a data availability statement. This statement should provide the following information, where applicable:

- Accession codes, unique identifiers, or web links for publicly available datasets
- A list of figures that have associated raw data
- A description of any restrictions on data availability

The RNA-seq data for this study are deposited in GEO with the accession code GSE126260. All other relevant data generated in this manuscript that support the findings of this study are available upon request from the authors.

Field-specific reporting

Please select the one below that is the best fit for your research. If you are not sure, read the appropriate sections before making your selection.

☒ Life sciences ☐ Behavioural & social sciences ☐ Ecological, evolutionary & environmental sciences

For a reference copy of the document with all sections, see nature.com/documents/nr-reporting-summary-flat.pdf

Life sciences study design

All studies must disclose on these points even when the disclosure is negative.

Sample size	Sample size varies and was determined based on anticipated scatter of the data, but formal power calculations were not done given the lack of previous comparable datasets from which to estimate variance.
Data exclusions	No data were excluded from the analyses.
Replication	When cells lines were used in experiments, at least two cell lines were used to reproduce the experiments/results. If replicates were used at least three biological or three technical replicates were included.
Randomization	Common and widely used hPSC and communcially available primary cell lines were used in this study. Biological replicates were chosen randomly.
Blinding	Blinding was performed for experiments when possible given different investigator performed experiment from investigator doing analysis (e.g. Q-PCR data); however, for some experiments blinding was not possible due to the same investigator performing and analyzing data.

Reporting for specific materials, systems and methods

We require information from authors about some types of materials, experimental systems and methods used in many studies. Here, indicate whether each material, system or method listed is relevant to your study. If you are not sure if a list item applies to your research, read the appropriate section before selecting a response.

Materials & experimental systems

n/a	Involved in the study
☐	☒ Antibodies
☐	☒ Eukaryotic cell lines
☒	☐ Palaeontology
☒	☐ Animals and other organisms
☐	☒ Human research participants
☒	☐ Clinical data

Methods

n/a	Involved in the study
☒	☐ ChIP-seq
☐	☒ Flow cytometry
☒	☐ MRI-based neuroimaging

Antibodies

Antibodies used	Please see Supplementary Table 2 for antibodies used in this study.
Validation	All antibodies were commercially purchased and validated against human epitopes as stated on the datasheet for each antibody and on the commercial website. We titrated each antibody to test the optimal dilution to specifically label the cells.

Eukaryotic cell lines

Policy information about cell lines

Cell line source(s)	Human iPSC line DF19-9-11T and ESC lines H1 and H9-TnnT2-GFP were used in this study (WiCell Research Institute, cell line nam e: iPS DF19-9-11T; WA01; H9-hTnnT2-pGZ-D2, respectively). Primary human fetal ventricular cardiac fibroblasts (hfV-CFs, Cell Applications, Inc., cryopreserved HCF, fetal: 306-05f, lot 2666.) and adult ventricular cardiac fibroblasts (haV-CFs, Lonza, NHCF-V, Human Cardiac Fibroblasts-Ventricular, CC-2904, lot 0000401462) and primary dermal fibroblasts (hDFs) (healthy donors, 020a and 023a lines, generated with informed consent as approved by UW-Madison Health Science IRB, protocol H-2008-0250, were used in this study.
Authentication	STTR testing was done for cells obtained from WiCell and other commercial testing to verify identity.
Mycoplasma contamination	All cell lines used in this study were tested negative for mycoplasma contamination. Please see the statement on page 20, line 441-442 on the manuscript.

Commonly misidentified lines (See ICLAC register)	None

Human research participants

Policy information about studies involving human research participants

Population characteristics	Subjects were recruited as healthy controls who are first degree relatives to proband with inherited heart disease.
Recruitment	Subject were recruited from UW-Madison Inherited Arrhythmia clinic population who expressed interested in volunteering a skin sample for basic research using iPS cells.
Ethics oversight	The protocol was approved by the UW-Madison Health Science IRB, protocol H-2008-0250.

Note that full information on the approval of the study protocol must also be provided in the manuscript.

Flow Cytometry

Plots

Confirm that:

☒ The axis labels state the marker and fluorochrome used (e.g. CD4-FITC).

☒ The axis scales are clearly visible. Include numbers along axes only for bottom left plot of group (a 'group' is an analysis of identical markers).

☒ All plots are contour plots with outliers or pseudocolor plots.

☒ A numerical value for number of cells or percentage (with statistics) is provided.

Methodology

Sample preparation	Please see the Flow cytometry method in the manuscript on page 23.
Instrument	Please see the Flow cytometry method in the manuscript on page 23, and supplementary figures and legends for flow cytometry gating strategies for instruments used for data collection.
Software	FlowJo v10
Cell population abundance	Please see the figures for flow cytometry for the abundance of the relevant cell populations and purity of the samples.
Gating strategy	Please see Supplementary Figure 1, 3, 5, 6, 7, 8b, 9b, 11, 13, 17 and the figure legends for the sequential gating strategies used in the flow cytometry.

☒ Tick this box to confirm that a figure exemplifying the gating strategy is provided in the Supplementary Information.
