# Supplementary material for: Functional cardiac fibroblasts derived from human pluripotent stem cells via second heart field progenitors
Source: Nat Commun. 2019 May 20;10:2238. doi: 10.1038/s41467-019-09831-5 (PMC6527555; doi:10.1038/s41467-019-09831-5)
Supplement: Supplementary file 4 — Description of Additional Supplementary Files [file 41467_2019_9831_MOESM4_ESM.pdf]

Description of Additional Supplementary Files

Supplementary Data 1.
List of genes expressed in hPSC-CFs and haV-CFs and their corresponding expression values measured by RNA-seq (TPM) with log fold-change hPSC-CF/haV-CF > 3
Supplementary Data 2.
List of genes expressed in haV-CFs and hPSC-CFs and their corresponding expression values measured by RNA-seq (TPM) with log fold-change haV-CF/hPSC-CF > 3
Supplementary Movie 1.
Confocal Z-scan series of immunolabeled extracellular collagen (green) and fibronectin (red) produced in high density hPSC-CF culture. Nuclei are labeled with Hoechst (blue).
Supplementary Movie 2.
Confocal Z-scan series of immunolabeled extracellular collagen (green) and fibronectin (red) produced in high density hfV-CF culture. Nuclei are labeled with Hoechst (blue).
Supplementary Movie 3.
Confocal Z-scan series of immunolabeled extracellular collagen (green) and fibronectin (red) produced in high density haV-CF culture. Nuclei are labeled with Hoechst (blue).
Supplementary Movie 4.
Confocal Z-scan series of immunolabeled extracellular collagen (green) and fibronectin (red) produced in high density hDF culture. Nuclei are labeled with Hoechst (blue).
Supplementary Movie 5.
Confocal Z-scan series of immunolabeled fibronectin (red) and α-smooth muscle actin (green) in permeabilized high density hPSC-CF cultures. Nuclei are labeled with Hoechst (blue).
Supplementary Movie 6.
Optical mapping membrane of voltage of the monolayers from 100%hPSC-CMs.
Supplementary Movie 7a.
Optical mapping membrane of voltage of the monolayers from co-culture of 90%hPSC-CMs and 10% hPSC-CFs.
Supplementary Movie 7b.
Optical mapping membrane of voltage of the monolayers from co-culture of 90%hPSC-CMs and 10% haV-CFs.
Supplementary Movie 7c.
Optical mapping of membrane voltage from monolayers with co-culture of 90%hPSC-CMs and 10%hDFs.
Supplementary Movie 8a.
Optical mapping of membrane voltage from monolayers with co-culture of 50%hPSC-CMs and 50%hPSC-CFs.
Supplementary Movie 8b.
Optical mapping of membrane voltage from monolayers with co-culture of 50%hPSC-CMs and 50%haV-CFs
Supplementary Movie 8c.
Optical mapping of membrane voltage from monolayers with co-culture of 50%hPSC-CMs and 50%hDFs.
